# Reply to Neunhaeuserer et al. Comment on “Bianco et al. Differences in Arrhythmia Detection Between Harvard Step Test and Maximal Exercise Testing in a Paediatric Sports Population. *J. Cardiovasc. Dev. Dis.* 2025, *12*, 22”

**DOI:** 10.3390/jcdd12060226

**Published:** 2025-06-13

**Authors:** Massimiliano Bianco, Fabrizio Sollazzo, Riccardo Pella, Saverio Vicentini, Samuele Ciaffoni, Gloria Modica, Riccardo Monti, Michela Cammarano, Paolo Zeppilli, Vincenzo Palmieri

**Affiliations:** 1 Unità Operativa Complessa di Medicina dello Sport e Rieducazione Funzionale, Fondazione Policlinico Universitario Agostino Gemelli IRCCS, Università Cattolica del Sacro Cuore, 00168 Rome, Italy; massimiliano.bianco@policlinicogemelli.it (M.B.); riccardo.pella94@gmail.com (R.P.); saverio.vicentini@gmail.com (S.V.); samuele.ciaffoni01@icatt.it (S.C.); riccardo.monti1@unicatt.it (R.M.); michela.cammarano01@gmail.com (M.C.); paolo.zeppilli@unicatt.it (P.Z.); vincenzo.palmieri@unicatt.it (V.P.); 2 Dipartimento di Neuroscienze, Università Cattolica del Sacro Cuore, 00168 Rome, Italy; gloria.modica@icatt.it

We have read the commentary on our article entitled ‘Differences in Arrhythmia Detection Between Harvard Step Test and Maximal Exercise Testing in a Paediatric Sports Population’ [1]. We are very grateful for the interest in our work as well as the relevance and constructive nature of the comments made [2]. It is indeed only through renewed attention to the methodologies used in the application of pre-participation screening to athletes that a significant improvement in the quality of the diagnostic tools used can be achieved. In this regard, we feel it is important to answer and better clarify some of the points you have commendably raised.

First of all, we would like to emphasize that in this work of ours, we never advocated the desire to assess arrhythmic prevalence in the general sports population. In fact, we are keenly aware that our center, performing a second- and third-level activity for the territory, encounters a higher prevalence of arrhythmic events than the general population. Nevertheless, the repeated evaluation of the same sample using the two different examination modalities allowed a more than reasonable comparison between the two types of tests in relation to the desired outcome, in this case, the occurrence of arrhythmia in two different test modalities. In essence, our study is exclusively a comparison between the two test modalities and is not intended to be a prevalence study in any way.

With regard to the reported maximality criteria, we would like to underline that they do not represent real exclusion criteria or a source of selection bias: our center has over forty years of experience in HST evaluation, and our main objective has always been to obtain a test that is as maximal as possible. In fact, we believe that regardless of the test mode, it is the fulfilment of maximality criteria that is the real strength of a correctly performed HST. This can be achieved by taking a few specific precautions:

- Instruct the athlete in advance that he/she must give his/her best performance for the entire duration of the test.
- Ensure continuous and consistent verbal encouragement is provided throughout the test by the physician/nurse and, in the case of very young athletes, a parent, if required.
- Extend the duration of the test must until at least one of the two criteria of maximal testing mentioned in our paper (i.e., Rate of Perceived Exertion (RPE) score ≥ 9 on the Borg Category Ratio 10 (CR-10) scale (ranging from 0 to 10) or a maximum heart rate > 85% of the expected maximum heart rate for age based on the Cooper formula (220 − age)) is fulfilled.

Accordingly, as reported in our results, the average duration of the HST was 191 ± 21 s (which is longer than 3 min), with a minimum of 180 s and a maximum of 300 s, in accordance with this goal.

In relation to the inclusion of different maximum ergometric test modes, such as treadmills and cycle ergometers, we consider the objection to be entirely legitimate, given that our aim would be precisely to compare different test modes. It should be noted, however, that the use of a treadmill does not constitute our standard operating procedure and is reserved for a very limited number of people, mainly those who are not tall enough to reach the pedals of a bicycle ergometer. This approach is deeply rooted in the European tradition of ergometric testing. Consequently, the number of tests conducted on the treadmill was minimal (only six), which makes this factor insignificant in terms of the overall statistical analysis. It is noteworthy that both types of tests require the criteria of maximality to be fulfilled, in accordance with the corresponding guidelines [3]. Furthermore, the literature does not currently provide any evidence of the two test modes being able to induce a different arrhythmic response, so that they can be used interchangeably for the clinical objective of the maximal ergometric test.

With regard to the issue of reproducibility, it was not included in the present study as it was deemed to be a somewhat distinct topic, thus exceeding the aim of our paper; however, the Cohen’s k was 0.475 for supraventricular beats and 0.552 for ventricular beats. This finding indicates moderate reproducibility between the two test modalities. However, it is important to note that this result may be misleading: even if we point out that an arrhythmic event occasionally does not manifest itself consistently in both test modes, there is still evidence that there is one test mode that indicates more arrhythmias between the two. Furthermore, the moderate reproducibility of arrhythmias can be partly explained by the fact that the majority of the sample did not present any evident cardiovascular disease. Indeed, it is generally accepted that the reproducibility of arrhythmias in repeated exercise testing is a criterion for the suspicion of structural heart abnormalities [4,5].

**Conclusions**

In conclusion, the most important point that may emerge from our work is the need for all actors involved in the pre-participation screening of athletes to try their best to get as close as possible to the goal of a maximal test throughout the HST. Based on the data and our experience, achieving this objective—which has been underestimated until now—could represent a major development direction, enhancing the effectiveness of future Italian guidelines for pre-participation screening in sports [6].
